# Tai Chi Chuan Auxiliary Training Systems in Health and Rehabilitation: Scoping Review

**DOI:** 10.2196/64207

**Published:** 2025-12-22

**Authors:** Hong Liu, Huibiao Li, Haoyu Huang, Jia Huang, Yanxin Zhang, Lidian Chen

**Affiliations:** 1 College of Rehabilitation Medicine, Fujian University of Traditional Chinese Medicine, Fuzhou, Fujian, China; 2 National-Local Joint Engineering Research Center of Rehabilitation Medicine Technology, Fujian University of Traditional Chinese Medicine, Fuzhou, Fujian, China; 3 Department of Exercise Sciences, The University of Auckland, Newmarket, Auckland, New Zealand; 4 The Institute of Rehabilitation Industry, Fujian University of Traditional Chinese Medicine, 1 Qiuyang West Road, Fuzhou, Fujian, 350122, China, 86 059122861815

**Keywords:** Tai Chi Chuan, human-computer interaction, physical activity, virtual reality, auxiliary training system, scoping review, PRISMA, Preferred Reporting Items for Systematic Reviews and Meta-Analyses

## Abstract

**Background:**

Tai Chi Chuan (TCC), often described as “moving meditation,” is a traditional Chinese mind-body exercise suitable for individuals of all ages. Mounting evidence demonstrates that TCC can improve physical functions, promote physical activity, and positively impact health and longevity. However, systematic learning is hindered by insufficient teaching resources, difficulties in imparting expertise, and learning environment constraints. TCC auxiliary training systems, an innovative means of human-computer interaction, provide a potential solution.

**Objective:**

This scoping review evaluates the research trends and clinical outcomes of TCC auxiliary training systems. Specifically, we compare the development tools, system design, and evaluation or validation processes used by different systems to guide future development in this research area.

**Methods:**

Following the PRISMA-ScR (Preferred Reporting Items for Systematic Reviews and Meta-Analyses extension for Scoping Reviews) guidelines, electronic databases (PubMed, Embase, Scopus, IEEE Xplore, and ACM Digital Library) were systematically searched for studies in English from 2014 to 2024. Two reviewers independently extracted the data and used an adapted version of the Santos evaluation criteria to evaluate the quality of the included studies. The included studies were qualitatively summarized with respect to system design and evaluation verification.

**Results:**

Among the 2202 identified studies, 34 studies met the inclusion criteria, of which 24 were rated as medium to high quality. Desktop-based applications dominate the TCC auxiliary training system environment, comprising 38% (13/34) of the selected studies. The hardware and software components of TCC auxiliary training systems vary depending on the development objectives. Regarding system design, 76% (26/34) addressed all groups, with only a minority focusing on specific populations. Interaction design in TCC auxiliary training commonly incorporates human-computer interaction technologies, such as tactile, action, visual, speech, and multimodal interaction. Clinical validation is necessary to implement this system in clinical practice. Most reviewed studies were validated, 6 underwent acceptability validation, 21 underwent feasibility validation, and only 2 virtual reality–based systems underwent clinical efficacy validation, demonstrating their effectiveness in improving cognitive abilities and motor functions in older adults.

**Conclusions:**

The TCC auxiliary training system is an innovative health intervention in a rapidly advancing field. This scoping review, the first undertaken on this topic, systematically synthesizes current evidence regarding its design, applications, research trends, and clinical outcomes, thereby establishing a comprehensive foundation to guide and inform future research. However, the current evidence still faces issues such as methodological inconsistencies, insufficient sample diversity, and a lack of long-term effectiveness validation, which limit its generalizability and effectiveness in widespread applications. Future research should place greater emphasis on standardized reporting, applicability to diverse populations, and foster ethical considerations and interdisciplinary collaboration. This will facilitate the widespread deployment of the TCC auxiliary training system and ensure its sustainable integration into the field of health intervention.

## Introduction

Physical inactivity is now recognized as the fourth leading cause of death and has been demonstrated to contribute to 40 chronic diseases [1]. Importantly, physical inactivity often plays an independent role as a direct cause of speeding the loss of cardiovascular and strength fitness, shortening healthspan, and lowering the age of onset of the first chronic disease, which in turn decreases quality of life, increases health care costs, and accelerates mortality risk [2]. In contrast, regular physical activity and exercise offer a wide range of health advantages, including enhanced physical fitness (notably in cardiorespiratory [3] and musculoskeletal capacities [4,5]), improved cardiometabolic health [6], cognitive function enhancement [7], mental health support [8], improved sleep quality [9], and an overall elevation in quality of life [10]. Furthermore, these activities contribute to reduced adiposity [11], lower all-cause mortality rates [12], and decreased health care expenditures [13,14] and positively impact healthy longevity and well-being [15].

Among various physical activities, Tai Chi, often described as “moving meditation” [16], is a traditional Chinese mind-body exercise suitable for individuals of all ages (including individuals with chronic diseases, cognitive impairment, or motor disorders) to support health [17-19]. Compared to brisk walking, Tai Chi is more effective at improving motor function—especially gait and balance—in individuals with Parkinson disease [20], reducing cardiovascular disease risk factors among adults with hypertension, and enhancing psychosocial well-being [21]. Compared to fitness walking, long-term Tai Chi exercise is more effective at improving global cognitive function in older adults with type 2 diabetes and mild cognitive impairment [22]. Compared to aerobic exercise, Tai Chi offers greater advantages in alleviating pain, anxiety, self-efficacy, and cognitive coping strategies in patients with fibromyalgia, and in reducing systolic blood pressure in individuals with prehypertension [23,24]. Compared to stretching exercise, Tai Chi is more effective at reducing the incidence of fall-related injuries, improving balance in older postmenopausal women who are cancer survivors [25], and decreasing injurious falls in older adults at high risk of falling [26,27]. Tai Chi is recommended by the “World Guidelines for Falls Prevention and Management for Older Adults: A Global Initiative” [28]. Moreover, compared to resistance and stretching exercises, Tai Chi can reduce balance impairments in patients with mild-to-moderate Parkinson disease, with additional benefits of improved functional capacity and reduced falls [29], and is now endorsed by the “Clinical Practice Guideline From the American Physical Therapy Association” [30].

Various Tai Chi Chuan (TCC) learning methods can accommodate different learning preferences and environments. Traditional methods include personalized instruction through one-on-one coaching and structured group classes, which provide direct feedback and hands-on guidance from experienced practitioners. Alternatively, self-directed learning through instructional videos, books, and online resources offers flexibility and accessibility, allowing individuals to study at their own pace. However, practitioners often find personal coaching cost-prohibitive, while self-study is monotonous and lacks the immediate corrective feedback necessary for mastering the subtleties of TCC, making it difficult to sustain over the long term. With the advent of virtual reality (VR) technology, the immersive, interactive, and imaginative characteristics of VR can set up a learning environment beyond the screen’s limitations, enhancing learners’ motivation and interest and producing ideal experiential results. The progress of VR technology has facilitated the emergence of the TCC learning system. A TCC auxiliary training system based on VR provides a safe, comfortable, and effective method to practice TCC through human-computer interactions with virtual interfaces. This system adapts to the needs and progress of each practitioner, providing a personalized rehabilitation experience and delivering comprehensive physical and psychological health benefits.

As early as 1997, Becker and Pentland [31] developed a virtual interactive TCC system to relieve stress in patients with cancer. The system uses a vision-based motion capture system to track the user’s head and hands, and the hidden Markov model was used to identify TCC movements and provide patient feedback. Since then, with the development of low-cost, highly cost-effective motion sensors such as Kinect and inertial sensors, more TCC auxiliary training systems based on VR and motion capture have been developed. Using such systems, TCC practitioners can follow the motions of the virtual coach to practice TCC and receive feedback from the system to adjust their practice in real time. The hardware of the TCC auxiliary training system based on VR usually includes TCC motion capture equipment, VR tools, and other commonly used basic computer hardware; the software requires game engines or modeling software to build VR scenes and virtual characters. The performance of the system depends on real-time bone tracking technology of the motion capture system, highly adaptive algorithms, good communication technology, and a real-time interactive VR environment. According to the existing research, this kind of TCC auxiliary training system can accelerate the learning process of practitioners and improve the quality of movement learning [32]. In addition, such a system design is more valuable for rehabilitating older adults and patients with chronic diseases from the point of view of long-term exercise [33,34].

Therefore, the TCC auxiliary training system could serve as a promising alternative to traditional and group TCC practices. It is particularly significant in the current health promotion and rehabilitation context, offering unprecedented opportunities to increase accessibility to rehabilitation training, enhance the coverage of health interventions across regions and cultures, and foster collaborative practices among different health care and community organizations.

Although studies have shown that TCC auxiliary training systems have potential advantages in improving motor skills, increasing training interest, and enhancing physical and mental health, gaps remain in their development and research. On one hand, there are significant differences in hardware (sensor types and accuracy), software (engines and algorithms), and human-computer interaction design across systems, leading to a lack of comparability between studies. On the other hand, clinical validation is insufficient, as most systems have only been tested in small-sample experimental settings and lack clinical trials. Furthermore, due to rapid technological advancements (such as motion capture and VR algorithms) and the increasing demand for rehabilitation in an aging population, no systematic review has specifically addressed the development, design, and effectiveness evaluation of TCC auxiliary training systems.

This review aims to fill this research gap by analyzing studies related to TCC auxiliary training systems from the past decade (2014‐2024), exploring the existing gaps in their development and research, assessing current trends and clinical outcomes, and providing a foundation for future research. This scoping review addresses the following research questions (RQs):

1. RQ1: To identify the development tools used across different systems, including the hardware and software used in their development.
2. RQ2: To describe system designs, including development environments, applications, and human-computer interactions, to identify research gaps and suggest design improvements for future design work.
3. RQ3: To identify the evaluation or verification of each system, including whether the system has been evaluated or verified and how it has been evaluated or verified, providing evidence for its efficacy in health promotion.
4. RQ4: To provide recommendations for further investigations in this research field.

This study aims to address these questions by summarizing the development tools, design, human-computer interaction, and clinical validation related to the “TCC auxiliary training system,” thus filling a gap in existing literature. Furthermore, it provides an integrated analytical framework that combines hardware, software, interaction design, and clinical validation, offering reference standards and benchmarks for future research and system development. Finally, it combines theoretical insights with practical considerations, providing evidence-based support for researchers, clinicians, and policymakers in clinical decision-making and the development of relevant health policies.

## Methods

### Literature Search Strategy

This protocol for the review was implemented following the PRISMA (Preferred Reporting Items for Systematic Reviews and Meta-Analyses) statement [35], which has been registered in the PROSPERO (International Prospective Register of Systematic Reviews) database under the ID CRD42024539375. Literature was retrieved from the following 5 databases: PubMed, Embase, Scopus, IEEE Xplore, and ACM Digital Library. The focus of this paper is the application, research trends, and clinical efficacy of the TCC auxiliary training system. There are many spellings of Medical Subject Headings terms for TCC, including Taiji, Tai Chi, TCC, and T’ai Chi. We modified the retrieval strategy according to different databases, and the search terms of all databases are provided in Multimedia Appendix 1. This study summarizes the studies published from January 2014 to May 2024.

### Inclusion and Exclusion Criteria

Studies eligible to be included in this review had to meet the following inclusion criteria: (1) studies describing a TCC training system, (2) the search language is limited to English, and (3) studies published in the past 10 years. The exclusion criteria were (1) systematic reviews or literature reviews, (2) books or study comments, (3) theses, (4) incomplete or short papers (eg, posters, tutorials, and technical reports), and (5) primary studies that are repeated.

### Study Selection Process

This search included journal and conference papers. The study screening process consisted of the following steps: (1) retrieving studies from January 2014 to May 2024, (2) screening titles and abstracts of the remaining studies after removing duplicates, and (3) reviewers reading the full texts and selecting studies according to the inclusion and exclusion criteria. If a journal study covers the content reported in the previous conference paper or degree thesis, the journal paper was given precedence over the conference paper and degree thesis.

### Assessment of Study Quality

After establishing the inclusion and exclusion criteria, the next step was to identify quality criteria to strengthen the extraction of quantitative and qualitative data for the synthesis and results analysis. We established a list of 5 quality standards (Table 1) based on Santos et al [36], aligning with the objectives of this review on TCC auxiliary training systems. Each study was rated by 2 authors (HuibiaoLi and HH) according to these 5 specific criteria. Finally, the final score of each researcher was sorted by Hong Liu, and any differences were checked. Any discrepancies between the raters were resolved through negotiation. Each criterion in the list is rated as follows: yes=1 point, no=0 points, and partial=0.5 points. The total score was derived by summing the scores of the questions, reflecting the overall quality of the included literature: 0‐2 (low quality), 3‐4 (medium quality), and 5 (high quality).

**Table 1.** Quality evaluation (QE) for this study.

Item	Description
QE1	The research scheme is described clearly, and the methods and techniques are explained.
QE2	The auxiliary training system of the TCC^a^ is clearly described.
QE3	A clear report on the virtual environment used.
QE4	A precise evaluation or verification of the system is carried out.
QE5	Detailed case study results are provided.

a TCC: Tai Chi Chuan.

### Data Extraction Process

After identifying eligible publications, all relevant data were collected in Microsoft Excel using a structured coding scheme. Data were extracted independently by 2 authors (Huibiao Li and HH). The collected variables included title, sample size, year, country, research methods and techniques, system description, type of virtual environment, system evaluation and validation, case study results, development tools, interaction design, and categories of TCC. Detailed descriptions were provided for development tools, system design, evaluation and validation methods, and clinical efficacy studies. Any discrepancies were resolved through discussion and re-evaluation of the relevant literature.

### Data Synthesis and Analysis

The data synthesis process used a narrative approach to analyze and present the findings of the included studies. An overview of the development tools used by different systems, descriptions of system designs, and evaluations or validations for each system were provided to facilitate cross-study comparisons. This study used summarized data wherever possible and followed the PRISMA-ScR (Preferred Reporting Items for Systematic reviews and Meta-Analyses extension for Scoping Reviews) guidelines.

## Results

### Database Search and Paper Lists

**Figure 1.**
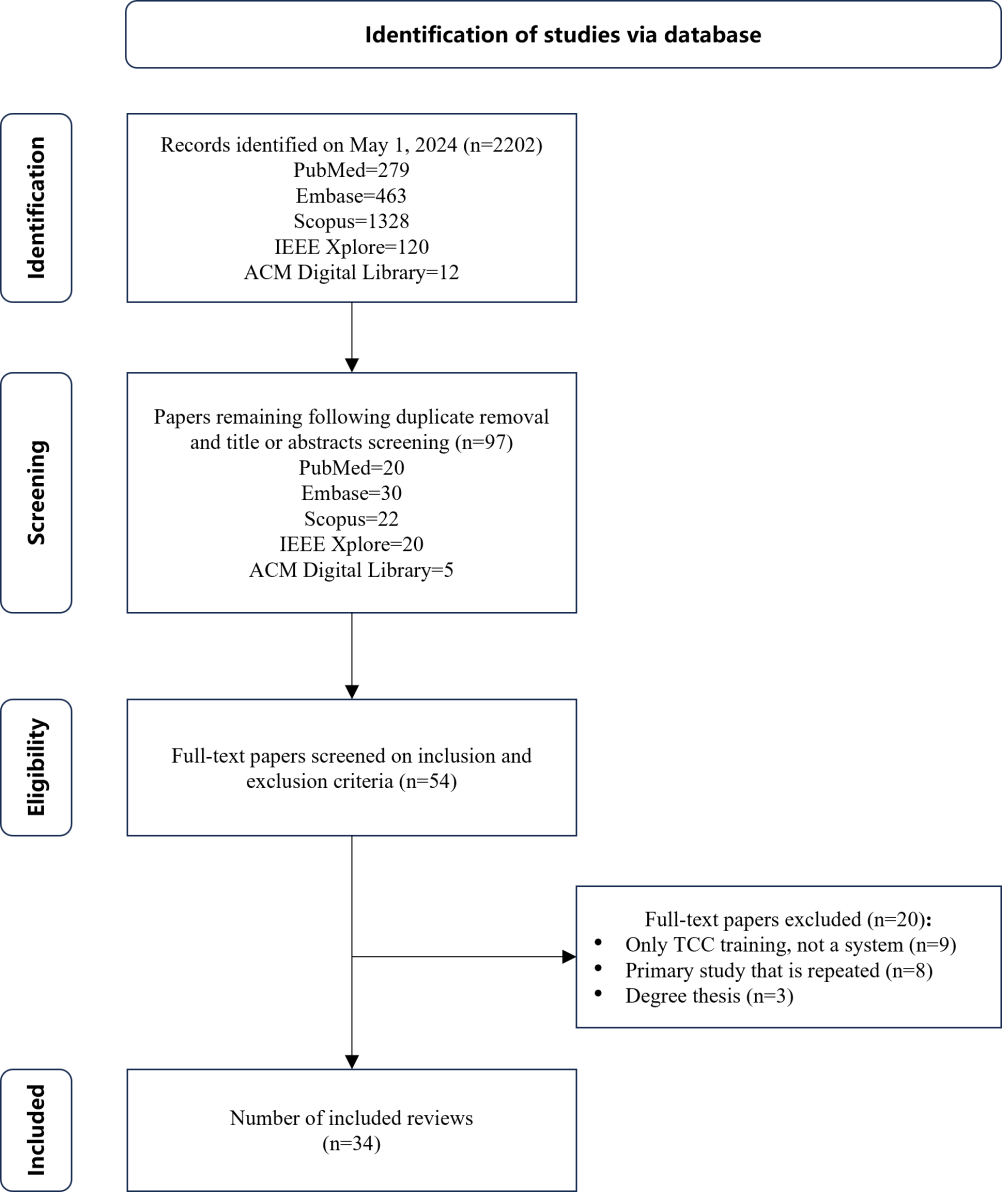
PRISMA (Preferred Reporting Items for Systematic Reviews and Meta-Analyses) flowchart of the results from the literature search. TCC: Tai Chi Chuan.

Figure 1 shows an overview of the study selection results at different stages. Of the 2202 studies retrieved through the search strategies, 34 papers were selected for inclusion in this review. Table 2 shows the quality of the studies included in the review. According to our predefined quality assessment criteria, 2 of the 34 studies included in the study were considered high quality, 22 were considered medium quality, and 10 were considered low quality. In summary, 94% (32/34) of the research schemes described the methods and techniques used, 82% (28/34) described the TCC auxiliary training system, 32% (11/34) reported the virtual environment used, 50% (17/34) evaluated or verified the system, and 41% (14/34) provided detailed case study results.

**Table 2.** Risk of bias assessment of the included studies.

Study	QE1^a^	QE2^b^	QE3^c^	QE4^d^	QE5^e^	Sum	Quality
Lee et al [37]	1	1	0	0	1	3	Medium
Chen [38]	1	1	0	1	0	3	Medium
Iwaanaguchi et al [39]	1	1	1	0	0	3	Medium
Bian et al [40]	1	0	1	0	1	3	Medium
Han et al [41]	1	1	1	1	0	4	Medium
Han et al [42]	1	1	1	0	0	3	Medium
Xue et al [43]	1	1	0	0	0	2	Low
Liang et al [44]	1	1	0	0	0	2	Low
Guimarães et al [45]	1	1	0	1	1	4	Medium
Delfa et al [46]	0	1	0	0	0	1	Low
Bian et al [47]	1	1	0	0	1	3	Medium
Hsieh et al [48]	1	0	0	0	1	2	Low
Lin et al [49]	1	1	0	1	1	4	Medium
Yu and Xiong [50]	1	1	0	0	0	2	Low
Kao et al [51]	1	1	1	1	1	5	High
Zhu et al [52]	1	0	1	1	1	4	Medium
Hung et al [53]	1	1	0	0	0	2	Low
Chen et al [32]	1	1	1	0	0	3	Medium
Kamel et al [54]	1	1	0	1	1	4	Medium
Chen et al [55]	1	0	0	1	1	3	Medium
Tharatipyakul et al [56]	1	0	0	0	0	1	Low
Liu et al [57]	1	1	1	0	0	3	Medium
Li and Wang [58]	1	1	0	0	0	2	Low
Jan et al [59]	1	1	1	1	0	4	Medium
Gao et al [60]	0	1	0	0	0	1	Low
Wei et al [61]	1	1	0	1	0	3	Medium
Li et al [62]	1	1	0	1	0	3	Medium
Li et al [63]	1	1	0	1	1	4	Medium
Kim et al [64]	1	0	1	1	1	4	Medium
Tian et al [65]	1	1	0	1	1	4	Medium
Wang et al [66]	1	1	0	0	0	2	Low
Wang and Deng [67]	1	1	0	1	0	3	Medium
Kanchanapaetnukul et al [68]	1	1	0	1	0	3	Medium
Tian et al [69]	1	1	1	1	1	5	High

a QE1: the research scheme is described clearly, and the methods and techniques are explained.

b QE2: the auxiliary training system of the Tai Chi Chuan (TCC) is clearly described.

c QE3: a clear report on the virtual environment used.

d QE4: a precise evaluation or verification of the system is carried out.

e QE5: detailed case study results are provided.

### Types of Publications

Table 3 shows the year, country (based on the research unit of the first author), and type of publication included in the review. In the research we included, 65% (22/34) of the studies were conference papers, and the remainder were journal papers. Since TCC is a traditional martial art originating in China, 76% (26/34) of the studies are from China.

**Figure 2.**
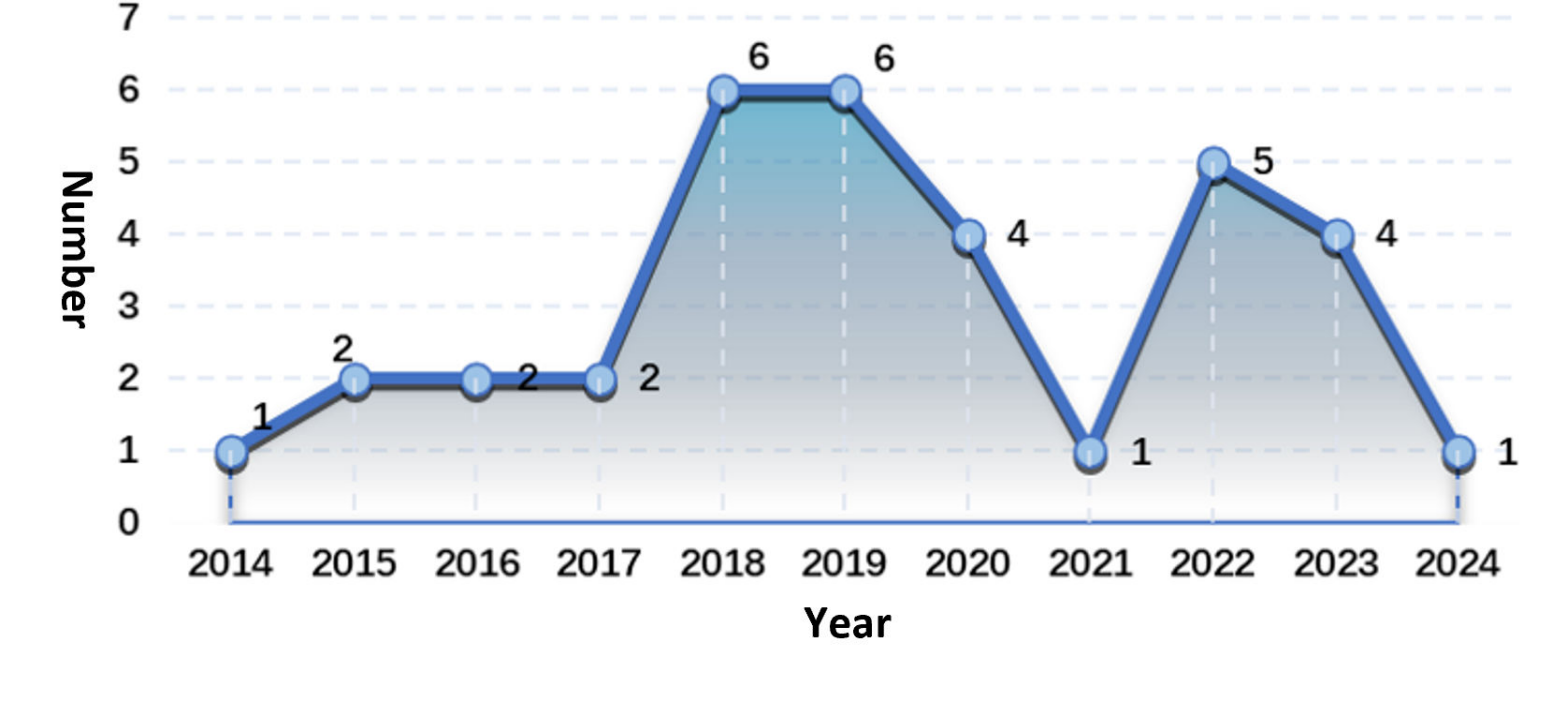
Time trend of research on Tai Chi Chuan (TCC) auxiliary training systems.

Figure 2 shows the temporal trend of the development of the TCC auxiliary training system from 2014 to 2024. This trend shows fluctuations in research over the past decade. In terms of time trends, research on TCC auxiliary training systems has been increasing in the past 10 years, with most system development research focusing on the period before and after the outbreak of the COVID-19 pandemic (59%, 20/34).

**Table 3.** Types of publications.

Publication type and year	China	Japan	United States	Korea	Singapore	Portugal	Thailand
Conference							
2014	[37]	—^a^	—^a^	—	—	—	—
2015	[38]	[39]	—	—	—	—	—
2016	[40,41]	—	—	—	—	—	—
2017	[42]	—	—	—	—	—	—
2018	[47,49]	—	—	—	—	[45,46]	—
2019	[51,53]	—	—	—	—	—	—
2020	[58]	—	—	—	[56]	—	—
2021	[59]	—	—	—	—	—	—
2022	[60-63]	—	—	—	—	—	—
2023	[66,67]	—	—	—	—	—	[68]
Journal							
2017	[43]	—	—	—	—	—	—
2018	[48]	—	[44]	—	—	—	—
2019	[32,52,54]	—	—	[50]	—	—	—
2020	[55,57]	—	—	—	—	—	—
2022	—	—	[64]	—	—	—	—
2023	[65]	—	—	—	—	—	—
2024	[69]	—	—	—	—	—	—

a Not available.

### RQ1: Comparison of Development Tools Used in Different TCC Auxiliary Training Systems

#### System Development Environment Design

This paper introduces the development tools of the TCC auxiliary training system from the hardware and software point of view. The hardware and software included in the 34 studies were analyzed. The hardware describes motion capture devices, VR tools, and other major hardware, as detailed in Table 4. The necessary devices, such as computers, keyboards, and mice, are not listed as hardware.

Most desktop application environments do not require hardware beyond commonly used devices, and keyboards and mice can be used to set up and simulate TCC virtual coaches in various software programs [70]. The presentation type of VR environment can be divided into immersive and nonimmersive types. Immersive VR uses a cave automatic virtual environment (CAVE), large screen projection, or helmet-mounted displays (HMDs) to present a VR environment, which can provide users with a strong sense of immersion [71]. In contrast, nonimmersive VR uses a desktop display system to present a VR environment, which does not provide a high degree of immersion [72]. Among the 34 studies we included, desktop-based environments are the most widely used in current TCC auxiliary training systems, accounting for 38% (13/34) of the selected studies. This is followed by HMD-based environments, which account for 26% (9/34). Web display and augmented reality (AR) system applications are less common, each representing 15% (5/34), while CAVE applications are the least common, accounting for 3% (1/34).

In the implementation tools of the VR environment for HMD systems, different types of devices are used, such as the Epson MOVERIO BT-200, Microsoft HoloLens, HTC VIVE, Oculus Quest, and Oculus Rift devices [32,39,41,42,51,52,57,59,64]. The AR system also uses a portable Microsoft HoloLens as an AR device to develop the TCC auxiliary training system [53].

In addition, the development purposes of TCC auxiliary training systems are different, and the hardware is also different. For example, the correct distribution of the center of gravity in TCC practice is essential, so they added plantar pressure–sensing devices to the TCC auxiliary training system to help practitioners perform accurate TCC movements with the correct center of gravity distribution [38,49,51]. Moreover, TCC exercises frequently incorporate metaphorical imagery. A developed TCC auxiliary training system, which uses drones to simulate gestures akin to “waving clouds,” can enhance the fluidity of movements [46].

**Table 4.** Development tools.

Study	Application environment	Motion capture	VR^a^ tools	Other	Software
Chen et al [32]	VR and HMD^b^ and CAVE^c^	Kinect	A 6-sided cubic screen and Oculus Rift	NR^d^	Unity3D
Iwaanaguchi et al [39]	HMD	MAC3D	Epson MOVERIO BT-200	NR	Maya and Unity3D
Bian et al [40]	VR	NR	NR	NR	NR
Han et al [42]	MR^e^-HMD	Parrot Bebop 2	Microsoft HoloLens	NR	Unity3D
Xue et al [43]	Desktop	Kinect	NR	NR	Unity3D
Lin et al [49]	Desktop	Kinect	NR	Pressure-sensing shoes	NR
Liang et al [44]	VR and AR^f^	Kinect	Oculus Rift	Hadoop cluster, GPU server, and Foot-pressure pad and Activator	Unity 3D, TensorFlow, Kinect Studio and Gesture recog. toolkit
Yu and Xiong [50]	Desktop	Kinect and Xsens MVN BIOMECH	NR	NR	Unity3D
Kao et al [51]	MR-HMD	Vicon	Microsoft HoloLens	Pressure-sensing insoles	Unity3D
Zhu et al [52]	HMD	Kinect	Google Cardboard and Smartphone	NR	Google VR SDK
Hung et al [53]	AR	Vicon and Sony SmartWatch 3	Microsoft HoloLens	NR	NR
Chen et al [55]	AR	Kinect	NR	NR	NR
Tharatipyakul et al [56]	Desktop	Logitech C922 Pro webcam	NR	NR	JavaScript
Liu et al [57]	HMD	Vicon and Noitom	HTC VIVE	NR	Maya and Unreal Engine 4
Li and Wang [58]	Desktop	Kinect	NR	NAO robot, STM32, DC deceleration motor, and RFP pressure sensor	Python, C language, and MATLAB
Gao et al [60]	Desktop	Kinect	NR	Screen display	NR
Guimarães et al [45]	Desktop, Web, and Mobile	Inertial Sensors	NR	Bluetooth® Low Energy	Unity3D
La et al [46]	Micro UAVs^g^	Qualisys	NR	Crazyflie 2.0s and LED	Python
Bian et al [47]	Desktop	NR	NR	Smartphone and Separate display	NR
Han et al [41]	AR-HMD	Leap Motion, Myo, and Chest Strap	Oculus Rift DK2	NR	Unity3D
Tian et al [65]	VR-HMD and MR-HMD	NR	NR	NR	Unity3D
Wang and Deng [66]	Web	Kinect	NR	Gyroscope, Accelerometer, Magnetometer, WIFI module, High-frequency filtering circuit, Signal conditioning circuit, A/D reference power circuit, E2PROM data storage circuit and Watchdog circuit	Unity3D
Wang and Deng [67]	Web	Kinect	NR	NR	Unity3D, HTML5, C language, Kinect SDK, and Microsoft Visual Studio 2010
Kanchanapaetnukul et al [68]	Desktop	Kinect	NR	NR	NR
Wei et al [61]	Web	Industrial camera	NR	NR	NR
Li et al [62]	Desktop	Kinect	NR	NR	NR
Li et al [63]	Web	NR	NR	NR	MySQL and PHP
Jan et al [59]	AR and OST-HMD	Webcam	Microsoft HoloLens	3-Axis Magnetic Sensor QMC5883L and Raspberry Pi	OpenPose and Lifting from the Deep
Lee et al [37]	Desktop	Kinect	NR	NR	NR
Tian et al [69]	TCG^h^, VR, and MR	NR	Pico Neo3 and Pico4	TP-LINK AX6000	Unity3D and Mirror Plugin
Kim et al [64]	VR	NR	Oculus Quest	NR	NR
Hsieh et al [48]	VR	Kinect	NR	NR	NR
Kamel et al [54]	Desktop	RGB-D	NR	Screen display	MakeHuman
Chen [38]	Desktop	NR	NR	Force sensor modules, microcontroller, and wireless data transmission module	NR

a VR: virtual reality.

b HMD: helmet-mounted display.

c CAVE: cave automatic virtual environment.

d NR: not reported.

e MR: mixed reality.

f AR: augmented reality.

g UAV: uncrewed aerial vehicle.

h TCG: traditional coach guidance.

The TCC virtual coach simulation is driven by motion data captured by human TCC experts. Commonly used motion capture systems can be divided into 2 categories: nonoptical systems and optical systems. Of the 34 studies we included, 3 used a combination of optical and nonoptical devices for accurate TCC motion capture; for example, Kinect and Xsens were used to capture the movement of each TCC practitioner [50]. Among the optical devices, the unmarked optical capture device, Kinect, is the most widely used, and 44% (15/34) of the TCC auxiliary training systems using Kinect as the motion capture device. Three studies used a tag-based Vicon system to capture TCC instructors’ movements [51,53,57].

In terms of software, Unity3D (David Helgason) is the most commonly used game engine for developing TCC auxiliary training systems, accounting for 38% (13/34) of the included studies. Unity3D is a cross-platform integrated game engine that mainly provides 3D animation, virtual scenes, physical computing, and other functions, which helps build a high-quality and high-fidelity virtual environment [73]. Maya (Autodesk, Inc.) is also used as 3D modeling software [39,57].

### RQ2: Description of the System Design for the TCC Auxiliary Training System

#### System Field Design

Table 5 describes the TCC auxiliary training system, the target population, and the TCC style used in the system. In the studies we included, 76% (26/34) of the TCC auxiliary training systems were aimed at all groups of people; only 5 systems were developed specifically for older adults [45,50,55,64,68], 1 system was developed for older adults with cognitive impairment [48], and 1 system was developed for people with dyskinesia [37].

**Table 5.** System field design.

Category	Study
Target population	
The TCC^a^ trainee	[32,38-44,46,47,49,51-54,56-63,65,66,69]
Older adults and older adults with cognitive impairment	[45,48,50,55,64,68]
People with movement disorders	[37]
The types of TCC	
24-form TCC and 18-form TCC	[39,40,43,44,54,55,60,66,68]
8-form TCC, Yang-style TCC, and Yang, Ye, Chen, or Wu-style TCC	[42,50,59,61-64,67,69]
Tai Chi Push Hands	[58]

a TCC: Tai Chi Chuan.

There are several variations in TCC, with the 24-form style being one of the most popular [39]. In developing TCC auxiliary training systems, approximately 24% (8/34) of the systems chose to capture the 24-form TCC demonstrated by TCC experts as a reference motion [39,40,43,44,54,55,60,66]. Six system developers implemented the Yang-style TCC in these systems [42,59,62-64,67], while 2 system developers chose the 8-form TCC [50,61]. In addition, a system developer selected the 18-form TCC as a reference [68]. Some developers opted to construct a Tai Chi Push Hands robotic system [69] or create a novel portable TCC group training system, which integrates various TCC forms [58].

#### System Interaction Design

Table 6 describes the human-computer interaction technologies commonly used in TCC auxiliary training systems, including tactile interaction, action interaction, visual interaction, speech interaction, VR input, and multimodal interaction technology.

**Table 6.** System interaction design.

Interactive technology and study	Algorithm
Motion interaction (posture recognition)	
Chen et al [32]	Quaternion-based with DTW^a^ extended from algorithms, curvature property in space curve extended from algorithms, and tensor decomposition–based extended from algorithms.
Xue et al [43]	DTW algorithm
Liang et al [44]	Approximate entropy or sample entropy
Lin et al [49]	DTW algorithm
Yu and Xiong [50], Hung et al [53], and Wang and Deng [66]	DTW algorithm
Li et al [62]	Deep-learning model
Kanchanapaetnukul et al [68]	BPNN^b^
Wei et al [61]	By applying YOLOv4^c^, TSDNN^d^, and PRNN^e^ to the obtained human body images, the system detects 17 human body keypoints.
Tian et al [65]	The average Euclidean distance between the hand positions of the standard movements and the learner’s movements across all frames.
Kamel et al [54]	CNN^f^ based on a set of algorithms that model high-level abstraction in data.
Jan et al [59]	DTW algorithm and Gaussian function–based similarity metric to compare the aligned signal.
Real-time visual interaction	
[38,43-49,51,52,55-57,59,60,62-65,68,69]	—^g^
Multimode interaction	
Tactile and Visual [44]	
Wearable sensors, Visual and Auditory [45]	—
Smartphone-based user interface and Visual [47]	—
Virtual keyboard technology, Motion, Speech and Visual [57]	—
Voice, Visual, Hidden panel and Hot key [59]	—
Voice, Visual and Background music playback [63]	—
Voice, rainbow trailing effects, hand models and particle tracks [65]	—
Visual, Touch control, Somatosensory Interaction, Phone interaction and Emotional interaction [67]	—
Button and Visual [69]	—

a DTW: dynamic time warping.

b BPNN: backpropagation neural network.

c YOLO: you only look once.

d TSDNN: time series deep neural network.

e PRNN: pose regression neural network.

f CNN: convolutional neural network.

g Not available.

Calculating the similarity of recorded TCC movements to a standard template is necessary to determine the interaction between TCC practitioners and the TCC auxiliary training system. In the current system design, the common algorithm for TCC posture recognition is the body posture recognition method based on template matching. Among these methods, the dynamic time warping (DTW) algorithm is mostly used [32,43,49,50,53,59,66], which calculates the proximity between the test sample and the standard sample by extending and shortening the time series, establishes a time-calibration matching path between the test sample and the standard sample, and identifies the path with the minimum cumulative distance between the 2 samples in the matching process as the optimal path. Developers have extended 2 methodological approaches to objectively evaluate TCC posture sequences. First, they enhanced a quaternion-based similarity assessment method, incorporating DTW and the curvature properties of spatial curves to improve motion sequence alignment. Second, they extended a tensor decomposition–based similarity assessment technique to capture multidimensional posture features for more accurate evaluation [32]. These extensions allow both quaternion-based similarity assessment with DTW and tensor decomposition–based similarity assessment to objectively evaluate TCC posture sequences.

In addition, some researchers used approximate entropy or sample entropy to measure the time series distance or dissimilarity between users and the TC master [44]. In recent years, the TCC action quality assessment has gradually been achieved through machine learning assessment models. These models use motion characteristics captured by computer vision combined with various neural networks, such as backpropagation neural networks, time series deep neural networks, convolutional neural networks, and pose regression neural networks, to facilitate TCC action recognition and assessment [54,61,62,68].

Excellent human-computer interaction design usually requires real-time feedback, and timely feedback can improve the user experience of a TCC auxiliary training system. Among the 34 studies included in this review, 62% (21/34) of the studies showed that the TCC auxiliary training system provides real-time visual feedback. Among the 21 studies that provided real-time visual feedback, 16 studies provided training feedback for TCC practitioners in the form of visual feedback, and 4 of them provided feedback in the form of scores [43,49,57,59]. In TCC practice, the correct center-of-gravity distribution of each motion is essential. Therefore, 3 system developments visualize the center-of-gravity distribution of practitioners practicing TCC via visual feedback to help practitioners perform TCC movements with the correct center of gravity [38,39,49,51].

TCC is a meditative practice where practitioners execute forms to unlock chi and allow it to flow within their bodies. Consequently, some researchers transformed TCC movements into a visual feedback form of energy flow, building a visual representation of the energy flows in the body [60]. Furthermore, TCC is a traditional therapeutic practice that combines breathing exercises with physical training; only 1 system visualizes the respiratory status of TCC practitioners in the form of visual feedback [39].

According to the literature review, only 9 TCC auxiliary training systems use multimodal interaction technology. For example, modern sensors, actuators, and VR or AR technologies can capture and reconstruct 4D motion behaviors, enabling seamless human-computer interaction [44]. The TCC auxiliary training system can also be operated via voice interaction, motion interaction, and virtual keyboard technologies, providing visual feedback of scores to practitioners [57]. Designers have also enhanced the interactive control experience by fixing a smartphone-based user interface to the user’s forearm, ensuring seamless interaction and positioning the smartphone as a key interaction tool [47,67]. Recently, a composite navigation module based on a unique environment has provided diverse interaction modes for TCC practitioners [65]. These modes encompass voice commands, rainbow trailing effects, hand models, and particle tracks to enrich the TCC experience. This integrated approach harnesses diverse methodologies and technologies, marking significant progress in optimizing user interaction and feedback mechanisms in the TCC auxiliary training system. These advancements promise to enhance TCC practices and potentially foster their widespread adoption.

### RQ3: Evaluation and Validation of the TCC Auxiliary Training System, With Evidence of Its Role in Health Promotion

This section summarizes the work conducted for evaluating or verifying each system according to the VR-core framework [74], a framework commonly used for evaluating VR systems, which includes evaluating system acceptability, feasibility, tolerability, and clinical efficacy.

#### Acceptability, Feasibility, and Tolerability

Table 7 describes the preliminary evaluation of the TCC auxiliary training system to clarify its acceptability, feasibility, and tolerability.

**Table 7.** Acceptability, feasibility, and tolerability of the Tai Chi Chuan (TCC) training system.

Domain and study	Participants	Result
Acceptability		
Chen et al [32]	Students (n=18)	ImmerTai in immersive environments is attractive to students and can enhance their learning experience significantly.
Yu and Xiong [50]	Older adults (n=41)	Participants thought this system was beneficial, positive, entertaining, with low privacy risk.
Kim et al [64]	Female participants (n=5)	VR^a^ provided participants with novel experiences while practicing TCC^b^, which they found valuable and enjoyable.
Jan et al [59]	Advanced TCC students (n=5)	The evaluation module clearly represented coach movements, the practice review UI^c^ was easy to use, and the module correctly identified errors and correct poses.
Li et al [63]	Students (n=246)	Students were more satisfied with “content selection,” “running speed,” and “login speed.”
Guimarães et al [45]	Older adults (n=8)	The exergame was a fun way to perform exercises, which they felt to be important to motivate people of their age.
Feasibility		
Chen et al [32]	Students (n=12)	The algorithm for assessing the movement quality of TCC is highly correlated with expert ratings in TCC.
Chen et al [32]	Students (n=18)	HMD^d^ > CAVE^e^ > PC^f^ for NoR^g^ (faster learning), CAVE > PC > HMD for MQS^h^ (better quality), and CAVE > PC > HMD for QSPR^i^ (higher learning efficiency).
Yu and Xiong [50]	Middle-aged and older participants (n=21)	Algorithm scores were comparable to the gold standard (experts’ ratings).
Iwaanaguchi et al [39]	Beginner (n=8)	The 2 controls were complementary to each other.
Han et al [42]	Participants (n=60)	Visualization of standard movement and a fixed augmented mirror.
Lin et al [49]	Beginner (n=14)	The TCC training system with weight transfer guidance and feedback benefited TCC beginners.
Zhu et al [52]	Volunteers (n=30)	The smoke-enhanced display effectively allows users to recognize and distinguish specific human motions.
	Volunteers (n=20)	Motion data presentation with smoke animation was beneficial and would help motivate long-term training.
Tharatipyakul et al [56]	Participants (n=12)	Trainer video and video with skeleton allowed participants to be significantly more accurate.
Kamel et al [54]	Volunteers (n=20)	iTai-Chi system can significantly improve learning outcomes of TCC learners.
Kamel et al [54]	Volunteers (n=60)	iTai-Chi > one-to-many tutorial > video watching (more accurately).
Kamel et al [54]	Volunteers (n=30)	Performance of older TCC practitioners was enhanced significantly using iTai-Chi.
Kamel et al [54]	Volunteers (n=40)	iTai-Chi overcomes the lack of previous knowledge.
Kamel et al [54]	Volunteers (n=130)	Practicing TCC with the system was exciting and increased motivation to practice continually.
Chen [38]	Participants (n=6)	Pressure-sensing shoes with visual feedback < without visual feedback (participants spent less time).
Hsieh et al [48]	Participants (n=60)	VR-based Tai Chi exercise provided protective effects for some cognitive and physical functions in older adults with cognitive impairment.
Tian et al [69]	Participants (n=36)	VR > MR^j^ > TCG (better effectiveness) and MR > TCG^j^ > VR (better social experience).
Kim et al [64]	Female participants (n=5)	VR Tai Chi benefits included mindfulness/enjoyment/physical exercise.
Jan et al [59]	Advanced TCC students (n=5)	Combining the camera and digital compasses > using only the camera (higher utility).
Wang and Deng [67]	A user	Students could improve their learning based on feedback results.
Tian et al [65]	Volunteers (n=9)	Significant improvements in the learners’ level of movement precision.
Wei et al [61]	Volunteers (n=4)	The system accurately assigned scores to users at different levels.
Lee et al [37]	A user with movement disorders	Significantly increased participants’ motivation for physical rehabilitation, improving exercise performance.
Li et al [63]	Students (n=246)	The system helped students learn TCC better (theory and skills).
Bian et al [47]	Students (n=40)	Improved learning experience/performance.
Guimarães et al [45]	Older adults (n=8)	Helped someone unfamiliar with exercises perform them correctly.
Han et al [41]	Participants (n=8)	All participants were guided to move their arms to perform maneuvers well.
Tolerability		
Tian et al [69]	Participants (n=36)	Despite the transitions between real and virtual worlds during the training, participants did not experience significant discomfort (VR/MR/TCG).
Kim et al [64]	Female participants (n=5)	In the virtual TCC program, participants had to bow their heads to view the location of their footsteps, which caused them to feel nausea.

a VR: virtual reality.

b TCC: Tai Chi Chuan.

c UI: user interface.

d HMD: helmet-mounted display.

e CAVE: cave automatic virtual environment.

f PC: personal computer.

g NoR: number of replays.

h MQS: motion quality score.

i QSPR: quality score per replay.

j MR: mixed reality.

Among the 34 studies included in this review, 6 studies evaluating user acceptability of the developed system showed promising results. The users surveyed thought that the TCC auxiliary training system based on VR is valuable and positive and can effectively improve their practice quality. In addition, 21 studies have analyzed the feasibility of the TCC auxiliary training system to determine existing system problems as quickly as possible to prepare for follow-up clinical trials. Researchers evaluated the feasibility of the TCC motion quality assessment algorithm and system in different VR environments, yielding favorable results [32]. In addition, the validity of the system’s assessment algorithm was verified, showing a high correlation with expert ratings [50]. Other researchers evaluated the TCC auxiliary training system under 2 control modes, demonstrating its feasibility and interoperability [39]. Feedback collected through interviews identified existing issues within the current system [42]. Furthermore, the TCC auxiliary training system with center-of-mass guidance and feedback functions is more beneficial for beginners. The smoke-enhanced display helps users effectively identify and differentiate specific body movements, facilitating TCC practice [49,52]. However, other researchers found that displaying virtual videos is also a feasible approach [56]. Finally, 2 studies specifically analyzed the tolerability of the TCC auxiliary training system. One study reported that participants did not experience significant discomfort when switching between real and virtual environments [69]. In contrast, another study observed that participants in virtual Tai Chi training experienced nausea due to the need to lower their heads to check foot positioning [64].

#### Clinical Efficacy

Regarding the clinical efficacy evaluation of the TCC auxiliary training system, only 2 studies align with the international working group’s recommendations [74]. In this study, 1 study randomly divided older adults into AR TCC auxiliary training and traditional TCC training groups. After 8 weeks of 30-minute training sessions, 3 times a week, the 3 balance function test scores improved in both groups. AR-assisted training with selected TCC movements, which are designed based on objective measurements of the practitioner’s capability, improved balance control and lower limb muscle strength at least as effectively as the complete sequence of traditional TCC exercises [55]. To explore the cognitive and physical effects of a VR-based Tai Chi (VRTC) exercise program on older adults with cognitive impairment, another study divided 60 older adults with cognitive impairment into clusters assigned to either the VRTC or the control group, and the intervention was conducted twice weekly for 6 months. The results suggested that the VRTC exercise program significantly protected abstract thinking and judgment, aerobic endurance, lower extremity endurance, balance, and gait speed, confirming the clinical efficacy of VRTC, as provided in Table 8.

**Table 8.** Clinical efficacy of the Tai Chi Chuan (TCC) training system.

Study	Chen et al [55]	Hsieh et al [48]
Design	RCT^a^	Quasi-randomized design study
Participants	Adults aged ≥65 years without any debilitating diseases (n=28)	Adults aged ≥65 years with cognitive impairment (n=60)
Comparisons (number of participants)	sTC^b^: using AR TCC^c^ training system to practice TCC (n=14)	VRTC: using AR TCC training system to practice TCC (n=14)
	tTC^d^: TCC masters guide the practice of TCC (n=14)	Control: TCC masters guide the TCC practice (n=14)
Exercise dose	30 minutes, 3 times per week for 8 weeks	VRTC^e^ group: 60-minute group session, twice weekly for 24 weeks Control group: no exercise or specific behavioral management training
Outcomes measurement	sTC: BBS^f^↑^g^, TUG^h^↑, FRT^i^↑, and muscle strength↑	VRTC group: 6MWT^j^↓^k^, 30-s STS^l^↓, FRT 5-m gait speed→^m^, and ABSTR^n^↑
Results	Equivalent	VRTC exercise yielded some cognitive and physical benefits
Trial registration	#1000087	103-1487B

a RCT: randomized controlled trial.

b sTC: selected Tai Chi.

c TCC: Tai Chi Chuan.

d tTC: traditional Tai Chi.

e VRTC: virtual reality–based Tai Chi.

f BBS: Berg Balance Scale.

g ↑: increased.

h TUG: timed up and go test.

i FRT: functional reach test.

j 6MWT: 6-minute walk test.

k ↓: decrease.

l 30-s STS: 30-second sit-to-stand test.

m →: no change.

n ABSTR: abstract thinking and judgment.

### RQ4: Future Directions for the Development of the TCC Auxiliary Training System

To enhance the innovation and foresight of the TCC auxiliary training system, we further explored its future development directions and proposed several potential technology integration approaches. These are aimed at addressing current challenges in personalized training, real-time feedback, and data analysis, thereby promoting the precision and intelligence of TCC training outcomes.

#### Integration of Artificial Intelligence (AI) With the TCC Auxiliary Training System

The rapid development of artificial intelligence (AI) has brought revolutionary changes to sports training and rehabilitation, particularly in areas such as motion recognition, posture optimization, and personalized training [75]. Among these, generative feedback and deep learning–based skeletal tracking, as key AI applications, show great potential in TCC-assisted training. The TCC training process requires precise posture control and dynamic coordination from practitioners. In this context, AI integration can provide more accurate feedback and guidance to the TCC auxiliary training system, improving both training effectiveness and efficiency.

Generative feedback is an AI-based technology that generates dynamic feedback by analyzing real-time motion data to provide targeted training suggestions [76]. In Tai Chi training, generative feedback analyzes the practitioner’s posture and movements in real time to offer personalized improvement suggestions. Compared to traditional manual guidance, generative feedback offers real-time, personalized, and repeatable characteristics, enabling practitioners to make subtle adjustments in each movement cycle, effectively reducing motion deviations and improving training quality. Deep learning–based skeletal tracking, using computer vision and deep learning algorithms, precisely tracks the movement of the human skeletal structure [77]. In Tai Chi training, deep learning–based skeletal tracking captures the practitioner’s full-body posture and joint movements in real time, generating accurate skeletal models. These skeletal models not only reflect an individual’s movement trajectories and posture changes but also reveal potential issues that may arise during the movements.

#### Integration of TCC Auxiliary Training Systems and Biofeedback Technology

Biofeedback technology, which involves real-time monitoring of physiological data and providing feedback, can present and regulate the practitioner’s physiological state to achieve training objectives [78-80]. In the application of TCC auxiliary training systems, biofeedback technology, particularly heart rate variability (HRV) and electroencephalography, provides significant potential for enhancing training outcomes. HRV, a physiological indicator of the regularity of heart rate fluctuations, can reflect the practitioner’s bodily responses in real time, particularly when facing varying levels of training intensity and load [80]. By monitoring HRV, training intensity and content can be dynamically adjusted to avoid the physiological burden caused by overtraining, thereby achieving a personalized training program. Changes in electroencephalography signals reflect psychological characteristics such as attention, relaxation state, and emotional fluctuations [81]. For TCC auxiliary training systems, real-time electroencephalography feedback serves as a crucial tool for users to adjust their mental state and optimize training outcomes. TCC, as a traditional exercise form that emphasizes mind-body coordination, particularly focuses on the practitioner’s internal concentration, breath control, and body movement coordination. With the integration of electroencephalography, TCC auxiliary training systems can detect when the practitioner’s brain is in an overly excited or anxious state and provide appropriate feedback to guide them in relaxation exercises such as deep breathing or meditation, helping them achieve an optimal mental state and thus enhancing training effectiveness [82].

#### Integration of the TCC Auxiliary Training System With Digital Twin Technology

Digital twin technology has rapidly advanced in recent years and is now widely applied across various fields, including smart manufacturing, health care, and sports training [83,84]. In the field of sports training, the application of digital twins enables precise simulation and prediction of an individual’s movement state and performance through virtual models. The integration of the digital twin with the TCC auxiliary training system is a novel design approach, promising enhanced precision and personalization in training while providing effective feedback on posture control and movement efficiency during exercise.

Posture twin uses detailed modeling of human movement to track actions in real time and provide optimization suggestions based on biomechanical principles. A core goal of TCC training is to improve balance and coordination through slow, fluid movements, which is particularly important for older adults. Posture twin not only tracks the balance status of Tai Chi practitioners in real time during each movement cycle but also combines gait analysis to assess gait symmetry, stability, and movement fluidity. In the future, with the development of digital twin technology, its application in TCC training will expand, opening new research directions for interdisciplinary integration in fields such as kinesiology and rehabilitation medicine.

In summary, the future development of the TCC auxiliary training system, integrated with advanced technologies such as AI, biofeedback, and digital twin, demonstrates great potential. Through the innovation and integration of these technologies, the system not only provides more personalized, real-time, and precise training feedback but also optimizes the physiological and psychological state of the trainee, thereby improving overall training effectiveness and efficiency. Future research can further deepen the application of these technologies, explore more interdisciplinary integration paths, and contribute to advancing the TCC auxiliary training system.

## Discussion

### Principal Findings

The results of this review indicate that most individuals can effectively engage in TCC training using assistive systems, which offer significant benefits for both physical and mental health. Existing studies show that the development tools for TCC auxiliary training systems are diverse, enabling the creation of training environments through various hardware and software combinations. The design of interaction modes and feedback systems effectively enhances participants’ engagement, learning interest, and training outcomes, without causing significant adverse effects. However, clinical validation of TCC auxiliary training systems remains insufficient. This necessitates future research to conduct longitudinal studies with standardized reporting to strengthen clinical validation. Finally, this study offers forward-looking insights by providing practical, design-oriented recommendations, such as integrating emerging technologies. These recommendations aim to guide future development, an area that has not been sufficiently explored in previous practice. Therefore, this review not only synthesizes existing evidence but also provides a strategic roadmap for designing more scalable and feasible TCC auxiliary training systems.

### Design of the TCC Auxiliary Training System

Immersive VR is principally used in developing the VR environment of existing TCC auxiliary training systems. The TCC assistant training system based on immersive VR has significant advantages in improving the learning speed of TCC practitioners, among which the HMD environment with the best VR experience has the fastest learning speed. In addition, compared with personal computers and HMD, the CAVE environment is more helpful for improving learning quality and increasing action learning efficiency [32]. This may be because the VR environment provided by HMD and CAVE becomes the visual reality of the practitioner without any hint of the physical environment. By using sensory information related to exercise, practitioners improve their attention to learning and motivation and, subsequently, the learning efficiency of TCC. The virtual scene design of mixed reality is also used in the current system design. In mixed reality, TCC practitioners can coexist with virtual coaches and interact in a mixed environment. After wearing a helmet, the practitioner can see many virtual coaches around them, and the TCC movements of the coach from different angles can be easily observed. Simultaneously, movements can be corrected over time through the augmented mirror [42]. In contrast, the AR design in the practice system is closer to the real-world scene, introducing computer-generated elements, including the use of common devices such as smartphones or tablets as viewing media, thereby enhancing the sensory experience of the natural environment. Therefore, existing research [55] shows that using an AR-based TCC auxiliary training system for TCC training can effectively improve the balance control of older adults and increase the muscle strength of the lower extremities.

Microsoft HoloLens is the most widely used VR tool in implementing VR environments. It accurately tracks TCC practitioners’ head movements in real time and offers immediate interactive experiences [42,51,53]. Its enhanced features and improved health services also cater to clinical rehabilitation and medical environments [85]. Second, the VR tool Oculus Rift has the characteristics of high resolution, a large field of view, low weight, easy setup, and easy access to good driver support [44]. However, it is essential to note that if the training system is to be applied to older adults or people with dysfunction, then the weight of HMD must be considered. In addition, although the use of HMD for TCC learning has achieved good learning results, we must consider that helmet displays are limited to a certain extent in the field of vision.

Kinects are widely adopted for motion capture due to their low cost, portability, and markerless capabilities, enabling data collection in diverse environments. They are particularly prevalent in VR training systems and are recognized as safe, effective, and feasible tools for rehabilitation in geriatric, neurological, and sports settings [50,86,87]. However, the disadvantage of Kinect-based systems is lower capture accuracy. There are 2 solutions in the current system design to solve this problem. One is to use the high-precision Vicon system as the motion acquisition equipment for the TCC coach, while the practitioner uses Kinect to capture the motion. This system design ensures the professionalism and accuracy of the coach action database and increases system portability, which is no longer limited by time and place, allowing it to be used more widely. Another method is to use a multimodal input combination. For example, based on Kinect, the plantar pressure sensor works together to obtain motion data from TCC practitioners and instantiates virtual images from practitioners as real-time input, effectively increasing the interactivity of the virtual environment [44,49,58]. Notably, the validity of the kinematic data recorded by Kinect should be improved. The book “Virtual Reality for Physical and Motor Rehabilitation” recommends camera placement to obtain the best motion tracking for upper limb applications. The Kinect camera should be located within 30×30 cm^2^, at a distance of 1.45 to 1.75 meters from the user, and 0.15 meters to either the left or right [88]. This study can provide a reference for system development and design in the future.

Another multimodal interaction uses wearable devices, Noitom and HMD, as action and voice inputs, resulting in natural voice interaction and gesture interaction, which creates a stronger sense of immersive experience and dramatically increases the practitioner’s sense of participation. Simultaneously, from the perspective of neural rehabilitation, this visual-auditory interaction can effectively improve the cognitive ability of users, especially attention and working memory [89]. With respect to current technology, achieving a complete multimodal interactive VR environment is not easy. The ultimate goal of human-computer interaction is to achieve seamless interaction as if the TCC coach and the practitioner were interacting face-to-face. Therefore, based on multimodal means including gestures, voice, and touch, we should also consider vision-based facial expression capture, mental state assessment, and wearable physiological index detection.

When the TCC auxiliary training system converts the TCC action of the practitioner into signal data that can be calculated and analyzed through multiple input devices, the algorithm is then used for segmenting, feature extraction, and classification of signal data. The most frequently mentioned classification algorithm is the DTW algorithm based on template matching. In the TCC auxiliary training system, the algorithm is mainly used for similarity matching. With the wide application of the algorithm, several researchers have innovated and improved it. A study used 8 bone vectors from human bones and body directions as input features and proposed a concise version of the DTW algorithm, which can further convert DTW distances into meaningful performance scores without requiring expert training data and experience [50]. The algorithm was effective and consistent compared to the expert score. However, the background of the 3 scoring experts was not introduced, so the results require further validation.

In recent years, machine learning has emerged as one of the forefront technologies in AI and is undergoing rapid development. Neural network models relevant to machine learning have found extensive applications across various domains, including video analysis and digital image processing. The TCC auxiliary training system has recently started using neural network models for human posture estimation. Some studies transformed the human posture estimation problem into a neural network–based skeletal joint regression problem [61,68]. Compared to conventional motion capture systems, another study proposed a TCC auxiliary training system based on pose estimation using convolutional neural networks, which demonstrated enhanced accuracy in estimating the postures of TCC practitioners [54].

The TCC auxiliary training system helps practitioners complete the required movements through continuous human-computer interactions. Therefore, after applying the algorithm to evaluate the collected motion data, it is necessary to provide feedback on motion quality to the practitioner in real time to improve the degree of participation. According to the literature, real-time visual feedback is the most widely used feedback strategy. Through real-time visual feedback, TCC practitioners can see themselves on the same screen as the instructor in the video [56], as well as the center of gravity distribution [39,49,51] and breathing status [39], which helps practitioners quickly improve their physical movements, enhance their interest in learning, and improve learning efficiency. In addition, some studies have designed 3 exaggerated real-time virtual visual cues to display the center of mass distribution. This exaggerated design enables practitioners to more easily observe the center of mass distribution of the virtual coach, providing useful visual feedback, particularly suited for beginners [51]. Simultaneously, exaggerated virtual sports design is more entertaining, increasing practitioner engagement in the practice process and improving virtual interaction performance. Introducing virtual smoke into the TCC training system to enhance the effect of motion display can encourage practitioners to practice longer, support sports memory, and aid memory retention [52,90].

With the advancement of technology, multimodal interaction has provided TCC practitioners with a more enriched and personalized learning experience. The feedback-based spatial path teaching method helps learners achieve better training outcomes through feedback and guidance. In addition, to emphasize training personalization, effects such as voice prompts, track settings, and arrow indicators are introduced for learners to choose, which improves training effectiveness and user participation [65]. To overcome the interactive limitations of traditional teaching methods and enable TCC learners to fully experience the joy of motion learning through natural interactions with the system, multimodal interaction via multiplatform browsers has been used to seamlessly and swiftly interact with the system, controlling the scenes and characters in the web and achieving effective learning objectives [67].

Notably, some researchers [91] believe that real-time feedback may be more effective for beginners, while terminal feedback is more suitable for skilled users. Therefore, combining real-time and terminal feedback can be considered in future system design, stimulating practitioners’ efforts, motivation, and persistence to some extent and potentially exceeding the goals achieved in current practice.

### TCC Auxiliary Training System Evaluation

In our opinion, the TCC auxiliary training system based on VR serves both as an auxiliary training system and a medical tool for rehabilitation exercises. However, the use of this system in clinical settings must be clinically validated. Of the studies we reviewed, only 2 studies clinically validated the designed system. Compared to traditional coach-guided training groups, the AR-based TCC auxiliary training system is effective for rehabilitation in older adults, and its clinical efficacy has been validated [55]. In addition, combining the VR-based TCC auxiliary training system can offer VR and TCC exercise programs for older adults with cognitive impairments. Compared to the control group maintaining regular physical activity, this system preserves cognitive and physical functions in older adults with cognitive impairments [48].

Although the results mentioned in the above "Clinical Efficacy" section are promising, these systems still lack robust research designs, rigorous measurement methods, and standardized, appropriate clinical outcome measures. This not only affects the external validity of the intervention effects but also limits the implementation of large-scale, multicenter randomized controlled trials to some extent. Particularly, in the context of rapid system iterations, the stability and generalizability of these systems in long-term clinical pathways remain unclear.

To advance the field, we recommend that future studies: first, adopt multicenter, randomized controlled trials with sufficient sample sizes, focusing on populations with high clinical value, such as older adults at high risk of falls, patients with Parkinson disease, and those with mild cognitive impairment. The control group may receive traditional rehabilitation training, health education, or VR experiences with placebo effects, ensuring clear attribution of the intervention effects. The intervention period should be at least 8 weeks, with a follow-up period of 3 to 6 months, and measurements taken at multiple time points to assess the maintenance effects of the intervention. Second, future studies should clearly define prioritized outcome measures in the research protocol and align them with the 4 dimensions of the VR core framework: acceptability, feasibility, tolerance, and clinical efficacy. For example, clinical trials targeting older adults may include primary outcomes such as balance or cognitive function, and secondary outcomes such as quality of life, lower limb strength, fall incidence, and the 4 dimensions of the VR core framework. Finally, the TCC system should be integrated into clinical rehabilitation pathways or telemedicine frameworks. It could be implemented in rehabilitation departments of tertiary hospitals, where the TCC system can serve as an adjunct module for physical therapy or occupational therapy in inpatient and outpatient rehabilitation processes. In community rehabilitation centers, TCC could be promoted as a group or individualized intervention to enhance its accessibility. In addition, remote medical platforms could integrate head-mounted displays, motion sensors, and cloud-based monitoring to enable real-time guidance and data feedback in home settings, allowing physicians and rehabilitation therapists to remotely assess progress and adjust training programs [92].

Despite the VR core framework recommending a comprehensive evaluation of VR-based interventions across 4 key domains (acceptability, feasibility, tolerance, and clinical efficacy), most of the included studies in this review did not report all these dimensions. This omission limits the ability to fully assess the value and applicability of the TCC auxiliary training system. The lack of acceptability and feasibility data hinders understanding of whether the intervention can be practically adopted in various settings, while the absence of tolerance data impedes a robust assessment of safety and user comfort. Without this information, even positive clinical efficacy results may overestimate the practical applicability of these systems. Potential reasons for this reporting gap include the early development stages of many systems, heterogeneous study designs, and a focus in most studies on efficacy outcomes rather than comprehensive evaluations. Future research should incorporate all 4 domains of the VR core framework into study protocols and report using standardized measures, leading to more balanced and generalizable conclusions regarding the efficacy and implementation potential of VR-assisted TCC training systems, with higher comparability across studies.

### Clinical Implication of the TCC Auxiliary Training System

In recent years, there have been numerous new findings regarding the clinical efficacy of TCC, with traditional TCC practices proving highly effective in reducing stress and promoting meditation or relaxation. The research findings of Kim et al [64] also demonstrated that virtual TCC practices can induce relaxation, reduce stress, and promote mental tranquility. This suggests that the virtual TCC auxiliary training system can serve as a mindfulness and meditation tool for promoting emotional well-being in older adults. In addition, TCC auxiliary training systems enable individuals to engage effortlessly and authentically in TCC practices, significantly reducing physical and psychological barriers. TCC auxiliary training systems can create both standing and sitting TCC practices [44,93]. Sitting TCC can reduce the pressure on the joints to the greatest extent and eliminate pain or fear caused by exercise. Therefore, sitting during TCC practice can make the practitioner’s mind more relaxed and peaceful. Moreover, the TCC auxiliary training system offers unique features such as engaging virtual environments, soothing background music, and a scoring system. These features are attractive and motivating for participants, facilitating the execution of movements and meditation techniques.

The TCC auxiliary training systems are suitable for people of all ages, including those with chronic diseases or dysfunction. Therefore, these systems are effective for home and community rehabilitation and can maintain the continuity of neural rehabilitation by overcoming social obstacles such as distance and cost, allowing practitioners to adjust their training schedule and exercise intensity. For practitioners, this is an entertaining and healthy way of life that positively impacts the mental health, physical health, self-esteem, and attention of older adults.

Notably, only 2 of the included studies explicitly assessed and reported clinical efficacy, highlighting a critical gap in the current evidence base. Clinical efficacy is the most direct indicator of the health benefits of the TCC auxiliary training system. Although other metrics, such as acceptability or feasibility, may be favorable, the lack of clinical efficacy data limits the ability to determine the real-world therapeutic value of these interventions. This scarcity may be attributed to the early development stage of most systems, small sample sizes, short intervention durations, and a primary focus on technical validation rather than patient-centered outcomes. Therefore, future research should prioritize well-powered, well-controlled trials that include validated clinical outcome measures as primary endpoints, while also conducting comprehensive assessments of acceptability, feasibility, and tolerability to provide an overall evaluation of intervention impact.

### Limitations

This review has several limitations. First, our search strategy included studies broadly related to the TCC auxiliary training system but did not systematically target specific diseases or populations. As a result, this review may not fully capture TCC auxiliary training systems tailored to specific diseases (eg, depression, anxiety, bipolar disorder, or post-traumatic stress disorder) or populations (eg, middle-aged individuals, older adults, or beginners). Future research should adopt more refined approaches to evaluate TCC auxiliary training systems targeting different diseases or populations, providing a deeper understanding of their effectiveness for these groups.

Second, the lack of standardized clinical efficacy assessment criteria is a major challenge in this field. The assessment tools and clinical indicators used in different studies are often inconsistent, leading to poor comparability and consistency of the findings. Clinical efficacy is typically assessed based on short-term effects, with a lack of long-term follow-up studies, making the evaluation of the TCC auxiliary training system’s efficacy and lasting impact incomplete. This limits the credibility of broader adoption and clinical application. A standardized evaluation system should be established for TCC auxiliary training systems to improve the reliability of assessing their therapeutic effects.

Third, this review found that most of the included studies were conducted in China, likely due to TCC’s origin and widespread practice in the country. This geographic concentration may limit the generalizability of our findings to populations in other cultural and health care contexts. Factors such as familiarity with TCC culture, local rehabilitation practices, and available technological infrastructure may influence user engagement and intervention outcomes. In addition, although our search was limited to English-language databases, the dominance of studies from a single country may introduce geographical bias. Future research should include multicenter trials conducted in different geographic regions to validate the applicability and effectiveness of TCC-based training systems in diverse cultural and health care settings.

Finally, while this review addresses ethical issues related to TCC auxiliary training systems to a lesser extent, empirical research examining the real-world ethical, social, and clinical impacts remains limited. The ethical impacts and accessibility challenges associated with deploying TCC auxiliary training systems are critical and cannot be overlooked. On one hand, while the application of body-tracking technology can provide accurate motion data and personalized feedback, it also raises significant concerns about privacy protection. TCC training systems use high-precision sensors and cameras to monitor practitioners’ movements, posture, and physiological states in real time, thereby increasing the risk of personal data breaches, particularly during data storage, transmission, and analysis. This issue is especially concerning older adults or vulnerable populations, who may not fully recognize the potential risks of data collection or how their data will be used, thereby complicating privacy protection. Ensuring data anonymization, legal and compliant use, and informed user consent is therefore crucial. Accordingly, the system should establish a robust data privacy protection mechanism to ensure that users can make informed and autonomous choices.

On the other hand, another accessibility challenge for TCC auxiliary training systems is their applicability to frail older adults. Frail older adults often face multiple physical, cognitive, and psychological challenges, such as poor motor coordination, slow response times, and difficulties understanding and operating complex technologies. While TCC, as a mild form of exercise, can improve the physical health of older adults, complex system operations and interfaces may impose a burden on some frail individuals. Furthermore, the precision of the personalized training system and the intensity of real-time feedback may need further adaptation and adjustment based on the physical conditions of older adults. To ensure that the TCC auxiliary training system benefits all groups, system design must account for the diverse needs of users with varying physical conditions, provide an intuitive interface, and reduce technical barriers through personalized features. This will not only help older users overcome technical adaptation challenges but also ensure the system’s sustainability and wide applicability in real-world use. Despite these limitations, this review provides a comprehensive overview of the application of TCC auxiliary training systems in the current context, identifies key research gaps, and offers feasible suggestions for future studies.

### Conclusion

This review provides a comprehensive evaluation of the design, application, research trends, and clinical effectiveness of TCC auxiliary training systems and offers recommendations for future development. We followed the PRISMA-ScR guidelines and analyzed 34 peer-reviewed studies published after 2014. This review addresses 4 key questions (RQs): development tools (RQ1), system design (RQ2), evaluation or validation (RQ3), and future development (RQ4). It emphasizes current development trends of TCC auxiliary training systems and outlines the design framework required for future advancements.

For RQ1, our findings indicate that development tools for TCC auxiliary training systems are diverse in both hardware and software. In terms of hardware, motion capture devices, VR tools, and AR technologies are widely used. In terms of software, Unity3D plays a leading role in system development, aiding in the creation of high-quality virtual training environments. However, further attention is needed to integrate various types of motion capture technologies and develop more immersive systems to enhance training effectiveness.

Regarding RQ2, we found that the design of TCC auxiliary training systems addresses certain demographic needs, with the 24-form Tai Chi style as the primary reference, incorporating various interaction modes and algorithms. However, further optimization is needed in integrating different interaction modes and considering accessibility to enhance training outcomes and user engagement.

For RQ3, the findings suggest that TCC auxiliary training systems show promising results in acceptability, feasibility, tolerability, and clinical efficacy. However, further optimization of system design is needed to address existing usability barriers and conduct large-scale longitudinal studies to strengthen clinical validation. Finally, for RQ4, future research could further optimize the integration of technologies and promote interdisciplinary collaboration to enhance the intelligence and precision of TCC auxiliary training systems.

## Supplementary material

10.2196/64207 Multimedia Appendix 1 Search strategy for PubMed, Embase, Scopus, IEEE Xplore, and the ACM Digital Library.

10.2196/64207 Checklist 1 PRISMA-ScR (Preferred Reporting Items for Systematic Reviews and Meta-Analyses extension for Scoping Reviews) checklist.

## Abbreviations

AI: artificial intelligence
AR: augmented reality
CAVE: cave automatic virtual environment
DTW: dynamic time warping
HMD: helmet-mounted display
HRV: heart rate variability
PRISMA: Preferred reporting items for systematic reviews and meta-analyses
PRISMA-ScR: Preferred Reporting Items for Systematic Reviews and Meta-Analyses extension for Scoping Reviews
RQ: research question
TCC: Tai Chi Chuan
VR: virtual reality
VRTC: virtual reality–based Tai Chi

## References

[1] Kerr NR, Booth FW (2022). Contributions of physical inactivity and sedentary behavior to metabolic and endocrine diseases. Trends Endocrinol Metab.

[2] Booth FW, Roberts CK, Thyfault JP, Ruegsegger GN, Toedebusch RG (2017). Role of inactivity in chronic diseases: evolutionary insight and pathophysiological mechanisms. Physiol Rev.

[3] Zhang T, Zhou R, Wang T, Xin Y, Liu X, Huang H (2023). Effects of traditional mind-body movement therapy on chronic cardiopulmonary dyspnoea: a systematic review and meta-analysis. Thorax.

[4] González-Rocha A, Mendez-Sanchez L, Ortíz-Rodríguez MA, Denova-Gutiérrez E (2022). Effect of exercise on muscle mass, fat mass, bone mass, muscular strength and physical performance in community dwelling older adults: systematic review and meta-analysis. Aging Dis.

[5] Mcleod JC, Currier BS, Lowisz CV, Phillips SM (2024). The influence of resistance exercise training prescription variables on skeletal muscle mass, strength, and physical function in healthy adults: an umbrella review. J Sport Health Sci.

[6] Isath A, Koziol KJ, Martinez MW (2023). Exercise and cardiovascular health: a state-of-the-art review. Prog Cardiovasc Dis.

[7] Northey JM, Cherbuin N, Pumpa KL, Smee DJ, Rattray B (2018). Exercise interventions for cognitive function in adults older than 50: a systematic review with meta-analysis. Br J Sports Med.

[8] Chen R, Guo Y, Kuang Y, Zhang Q (2024). Effects of home-based exercise interventions on post-stroke depression: a systematic review and network meta-analysis. Int J Nurs Stud.

[9] Memon AR, Gupta CC, Crowther ME, Ferguson SA, Tuckwell GA, Vincent GE (2021). Sleep and physical activity in university students: a systematic review and meta-analysis. Sleep Med Rev.

[10] Rijal A, Nielsen EE, Adhikari TB (2023). Effects of adding exercise to usual care in patients with either hypertension, type 2 diabetes or cardiovascular disease: a systematic review with meta-analysis and trial sequential analysis. Br J Sports Med.

[11] van Baak MA, Mariman ECM (2023). Obesity-induced and weight-loss-induced physiological factors affecting weight regain. Nat Rev Endocrinol.

[12] Ekelund U, Tarp J, Steene-Johannessen J (2019). Dose-response associations between accelerometry measured physical activity and sedentary time and all cause mortality: systematic review and harmonised meta-analysis. BMJ.

[13] Mattli R, Farcher R, Syleouni ME (2020). Physical activity interventions for primary prevention in adults: a systematic review of randomized controlled trial-based economic evaluations. Sports Med.

[14] Oldridge N, Taylor RS (2020). Cost-effectiveness of exercise therapy in patients with coronary heart disease, chronic heart failure and associated risk factors: a systematic review of economic evaluations of randomized clinical trials. Eur J Prev Cardiol.

[15] D’Onofrio G, Kirschner J, Prather H, Goldman D, Rozanski A (2023). Musculoskeletal exercise: its role in promoting health and longevity. Prog Cardiovasc Dis.

[16] Tai Chi and your health. News in Health, National Institutes of Health.

[17] Choo YT, Jiang Y, Hong J, Wang W (2020). Effectiveness of Tai Chi on quality of life, depressive symptoms and physical function among community-dwelling older adults with chronic disease: a systematic review and meta-analysis. Int J Nurs Stud.

[18] Zheng G, Liu F, Li S, Huang M, Tao J, Chen L (2015). Tai Chi and the protection of cognitive ability: a systematic review of prospective studies in healthy adults. Am J Prev Med.

[19] Mak MK, Wong-Yu IS, Shen X, Chung CL (2017). Long-term effects of exercise and physical therapy in people with Parkinson disease. Nat Rev Neurol.

[20] Li G, Huang P, Cui SS (2022). Mechanisms of motor symptom improvement by long-term Tai Chi training in Parkinson’s disease patients. Transl Neurodegener.

[21] Chan AWK, Chair SY, Lee DTF (2018). Tai Chi exercise is more effective than brisk walking in reducing cardiovascular disease risk factors among adults with hypertension: a randomised controlled trial. Int J Nurs Stud.

[22] Chen Y, Qin J, Tao L (2023). Effects of Tai Chi Chuan on cognitive function in adults 60 years or older with type 2 diabetes and mild cognitive impairment in China. JAMA Netw Open.

[23] Wang C, Schmid CH, Fielding RA (2018). Effect of tai chi versus aerobic exercise for fibromyalgia: comparative effectiveness randomized controlled trial. BMJ.

[24] Li X, Chang P, Wu M (2024). Effect of Tai Chi vs aerobic exercise on blood pressure in patients with prehypertension: a randomized clinical trial. JAMA Netw Open.

[25] Winters-Stone KM, Horak F, Dieckmann NF (2023). GET FIT: a randomized clinical trial of Tai Ji Quan versus strength training for fall prevention after chemotherapy in older, postmenopausal women cancer survivors. J Clin Oncol.

[26] Li F, Harmer P, Fitzgerald K (2018). Effectiveness of a therapeutic Tai Ji Quan intervention vs a multimodal exercise intervention to prevent falls among older adults at high risk of falling: a randomized clinical trial. JAMA Intern Med.

[27] Li F, Harmer P, Eckstrom E, Fitzgerald K, Chou LS, Liu Y (2019). Effectiveness of Tai Ji Quan vs multimodal and stretching exercise interventions for reducing injurious falls in older adults at high risk of falling: follow-up analysis of a randomized clinical trial. JAMA Netw Open.

[28] Montero-Odasso M, van der Velde N, Martin FC (2022). World guidelines for falls prevention and management for older adults: a global initiative. Age Ageing.

[29] Li F, Harmer P, Fitzgerald K (2012). Tai Chi and postural stability in patients with Parkinson’s disease. N Engl J Med.

[30] Osborne JA, Botkin R, Colon-Semenza C (2022). Physical therapist management of Parkinson disease: a clinical practice guideline from the American Physical Therapy Association. Phys Ther.

[31] Becker DA, Pentland A (1997). Using a virtual environment to teach cancer patients t’ai chi, relaxation and self-imagery. https://tinyurl.com/588samwc.

[32] Chen X, Chen Z, Li Y (2019). ImmerTai: immersive motion learning in VR environments. J Vis Commun Image Represent.

[33] Arlati S, Colombo V, Spoladore D (2019). A social virtual reality-based application for the physical and cognitive training of the elderly at home. Sensors (Basel).

[34] Choi SD, Guo L, Kang D, Xiong S (2017). Exergame technology and interactive interventions for elderly fall prevention: a systematic literature review. Appl Ergon.

[35] Liberati A, Altman DG, Tetzlaff J (2009). The PRISMA statement for reporting systematic reviews and meta-analyses of studies that evaluate health care interventions: explanation and elaboration. PLoS Med.

[36] Santos A dos, Delamaro ME, Nunes FLS (2013). The relationship between requirements engineering and virtual reality systems: a systematic literature review.

[37] Lee JD, Hsieh CH, Lin TY (2014). A Kinect-based Tai Chi exercises evaluation system for physical rehabilitation.

[38] Chen YC, Chen YC, Kao PY, Lu KY, Wei SY, Hung YP (2015). Pressure sensing insoles for learning Tai-Chi Chuan.

[39] Iwaanaguchi T, Shinya M, Nakajima S, Shiraishi M (2015). Cyber Tai Chi - CG-based video materials for Tai Chi Chuan self-study.

[40] Bian Y, Yang C, Guan D (2016). Effects of pedagogical agent’s personality and emotional feedback strategy on Chinese students’ learning experiences and performance. CHI ’16: Proceedings of the 2016 CHI Conference on Human Factors in Computing Systems.

[41] Han PH, Chen K, Hsieh CH, Huang YJ, Hung YP (2016). AR-arm: augmented visualization for guiding arm movement in the first-person perspective. AH ’16: Proceedings of the 7th Augmented Human International Conference 2016.

[42] Han P, Chen Y, Zhong Y, Wang H, Hung Y (2017). My Tai-Chi coaches: an augmented-learning tool for practicing Tai-Chi Chuan. AH ’17: Proceedings of the 8th Augmented Human International Conference.

[43] Zhihong X, Liying Z, Zhenhua C, Haozhi Z, Chunhui Y (2017). Research of Tai-Chi-Chuan auxiliary training system based on Kinect. Journal of Hebei University of Science and Technology.

[44] Liang Y, Wu D, Ledesma D, Davis C, Slaughter R, Guo Z (2018). Virtual Tai-Chi system: a smart-connected modality for rehabilitation. Smart Health (2014).

[45] Guimarães V, Pereira A, Oliveira E, Carvalho A, Peixoto R (2018). Design and evaluation of an exergame for motor-cognitive training and fall prevention in older adults. Proceedings of the 4th EAI International Conference on Smart Objects and Technologies for Social Good.

[46] Delfa J, Jarvis R, Khot RA, Mueller F (2018). Tai Chi in The Clouds: using micro UAV’s to support Tai Chi practice. CHI PLAY ’18 Extended Abstracts: Proceedings of the 2018 Annual Symposium on Computer-Human Interaction in PLAY Companion Extended Abstracts.

[47] Bian Y, Yang C, Zhou C (2018). Exploring the weak association between flow experience and performance in virtual environments.

[48] Hsieh CC, Lin PS, Hsu WC (2018). The effectiveness of a virtual reality-based Tai Chi exercise on cognitive and physical function in older adults with cognitive impairment. Dement Geriatr Cogn Disord.

[49] Lin H, Han P, Lu K (2018). Stillness moves: exploring body weight-transfer learning in physical training for Tai-Chi exercise. MMSports’18: Proceedings of the 1st International Workshop on Multimedia Content Analysis in Sports.

[50] Yu X, Xiong S (2019). A dynamic time warping based algorithm to evaluate Kinect-enabled home-based physical rehabilitation exercises for older people. Sensors (Basel).

[51] Kao PY, Han PH, Jan YF, Yang ZW, Li CH, Hung YP (2019). On learning weight distribution of Tai Chi Chuan using pressure sensing insoles and MR-HMD.

[52] Zhu L, Wang Z, Wang Y, Song A, Potel M (2019). Follow the smoke: immersive display of motion sata with synthesized smoke. IEEE Comput Graph Appl.

[53] Hung Y, Kao P, Jan Y, Li C, Chang C, Han P (2019). ICS 2018: New Trends in Computer Technologies and Applications.

[54] Kamel A, Liu B, Li P, Sheng B (2019). An investigation of 3D human pose estimation for learning Tai Chi: a human factor perspective. Int J Hum-Comput Interact.

[55] Chen PJ, Penn IW, Wei SH, Chuang LR, Sung WH (2020). Augmented reality-assisted training with selected Tai-Chi movements improves balance control and increases lower limb muscle strength in older adults: a prospective randomized trial. J Exerc Sci Fit.

[56] Tharatipyakul A, Choo KTW, Perrault ST (2020). Pose estimation for facilitating movement learning from online videos. AVI ’20: Proceedings of the 2020 International Conference on Advanced Visual Interfaces.

[57] Liu J, Zheng Y, Wang K, Bian Y, Gai W, Gao D (2020). A real-time interactive Tai Chi learning system based on VR and motion capture technology. Procedia Comput Sci.

[58] Li Q, Wang S (2020). Design of Tai-Chi push-hands robot control system and construction of visual platform.

[59] Jan YF, Tseng KW, Kao PY, Hung YP (2021). Augmented Tai-Chi Chuan practice tool with pose evaluation.

[60] Gao Z, Wang A, Hui P (2022). Meditation in motion: interactive media art visualization based on ancient Tai Chi Chuan [Abstract]. MM ’22: Proceedings of the 30th ACM International Conference on Multimedia.

[61] Wei C, Wen J, Bi R (2022). Online 8-form Tai Chi Chuan training and evaluation system based on pose estimation.

[62] Li J, Hu H, Xing Q, Wang X, Li J, Shen Y (2022). Tai chi action quality assessment and visual analysis with a consumer RGB-d camera.

[63] Li Y, Yuan T, Yu K (2022). Design and implementation of taijiquan learning system based on PHP+ MYSQL.

[64] Kim J, Kim Y, Chang PS, Min Oh S, Han S (2022). A pilot study of virtual reality (VR) Tai Chi program on mental health among older adults during the COVID-19 pandemic. Am J Health Behav.

[65] Tian F, Zou J, Li K, Li Y (2023). Kung Fu Metaverse: a movement guidance training system. IEEE Trans Learning Technol.

[66] Wang L, Deng W (2023). Research on the auxiliary training system of tai chi fitness qigong based on computer 3D image vision technology.

[67] Wang L, Deng W (2023). Research on tai chi APP simulation system based on computer virtual reality technology.

[68] Kanchanapaetnukul S, Aunkaew R, Charernmool P, Daoudi M, Saraubon K, Visutsak P (2023). Tai chi exercise posture detection and assessment for the elderly using BPNN and 2 kinect cameras.

[69] Tian F, Ni S, Zhang X (2024). Enhancing Tai Chi training system: towards group-based and hyper-realistic training experiences. IEEE Trans Vis Comput Graph.

[70] Zhu W, Fan X, Zhang Y (2019). Applications and research trends of digital human models in the manufacturing industry. Virtual Reality & Intelligent Hardware.

[71] Gürerk Ö, Bönsch A, Kittsteiner T, Staffeldt A (2019). Virtual humans as co-workers: a novel methodology to study peer effects. J Behav Exp Econ.

[72] Paravizo E, Braatz D (2019). Using a game engine for simulation in ergonomics analysis, design and education: an exploratory study. Appl Ergon.

[73] Paravizo E, Braatz D (2018). Proceedings of the 20th Congress of the International Ergonomics Association (IEA 2018) IEA 2018 Advances in Intelligent Systems and Computing.

[74] Birckhead B, Khalil C, Liu X (2019). Recommendations for methodology of virtual reality clinical trials in health care by an international working group: iterative study. JMIR Ment Health.

[75] Aboueldahab A, Damaschi G, D’Addario M, Steca P (2025). Exploring young adults’ attitudes toward AI-driven mHealth apps: qualitative study. JMIR Hum Factors.

[76] Nairn B, Tsakanikas V, Gordon B (2025). Smart wearable technologies for balance rehabilitation in older adults at risk of falls: scoping review and comparative analysis. JMIR Rehabil Assist Technol.

[77] Cerfoglio S, Ferraris C, Vismara L (2024). Estimation of gait parameters in healthy and hemiplegic individuals using Azure Kinect: a comparative study with the optoelectronic system. Front Bioeng Biotechnol.

[78] Pak SS, Janela D, Freitas N (2023). Comparing digital to conventional physical therapy for chronic shoulder pain: randomized controlled trial. J Med Internet Res.

[79] Seong S, Kim H, Cho Y (2025). Impact of virtual reality-based biofeedback on sleep quality among individuals with depressive symptoms, anxiety symptoms, or both: 4-week randomized controlled study. J Med Internet Res.

[80] Ferreira S, Rodrigues MA, Mateus C, Rodrigues PP, Rocha NB (2025). Interventions based on biofeedback systems to improve workers’ psychological well-being, mental health, and safety: systematic literature review. J Med Internet Res.

[81] Lai YJ, Chiu HY, Wu KC, Chang CW (2025). Diaphragmatic breathing interfaces to promote relaxation for mitigating insomnia: pilot study. JMIR Serious Games.

[82] Steen JP, Kannan V, Zaidi A, Cramer H, Ng JY (2024). Mind-body therapy for treating fibromyalgia: a systematic review. Pain Med.

[83] Drummond D, Gonsard A (2024). Definitions and characteristics of patient digital twins being developed for clinical use: scoping review. J Med Internet Res.

[84] Vallée A (2024). Envisioning the future of personalized medicine: role and realities of digital twins. J Med Internet Res.

[85] Palumbo A (2022). Microsoft HoloLens 2 in medical and healthcare context: state of the art and future prospects. Sensors (Basel).

[86] Ayed I, Ghazel A, Jaume-I-Capó A, Moyà-Alcover G, Varona J, Martínez-Bueso P (2019). Vision-based serious games and virtual reality systems for motor rehabilitation: a review geared toward a research methodology. Int J Med Inform.

[87] Molhemi F, Monjezi S, Mehravar M (2021). Effects of virtual reality vs conventional balance training on balance and falls in people with multiple sclerosis: a randomized controlled trial. Arch Phys Med Rehabil.

[88] Levin MF, Deutsch JE, Kafri M, Liebermann DG (2014). Virtual Reality for Physical and Motor Rehabilitation.

[89] Wang D, Zheng Y, Li T, Peng C, Wang L, Zhang Y (2018). Multi-modal human-machine interaction for human intelligence augmentation. Sci Sin-Inf.

[90] Gielniak MJ, Thomaz AL (2012). Enhancing interaction through exaggerated motion synthesis. HRI ’12: Proceedings of the seventh annual ACM/IEEE international conference on Human-Robot Interaction.

[91] Timmermans AAA, Seelen HAM, Willmann RD, Kingma H (2009). Technology-assisted training of arm-hand skills in stroke: concepts on reacquisition of motor control and therapist guidelines for rehabilitation technology design. J Neuroeng Rehabil.

[92] Triantafyllidis A, Segkouli S, Zygouris S (2023). Mobile app interventions for Parkinson’s disease, multiple sclerosis and stroke: a systematic literature review. Sensors (Basel).

[93] Lin TY, Hsieh CH, Lee JD (2013). Kinect-based system for physical rehabilitation: utilizing tai chi exercises to improve movement disorders in patients with balance ability.

