# Supplementary material for: Tai Chi Chuan Auxiliary Training Systems in Health and Rehabilitation: Scoping Review
Source: J Med Internet Res. 2025 Dec 22;27:e64207. doi: 10.2196/64207 (PMC12723366; doi:10.2196/64207)
Supplement: Multimedia Appendix 1 [file jmir-v27-e64207-s001.docx]

**Appendix 1. Literature Search Strategy**

| **Database** | **Search Terms** | **Hits** |
| --- | --- | --- |
| PubMed | #1 "Tai ji"[MeSH Terms] OR "Tai-ji"[Title/Abstract] OR " Tai Chi"[Title/Abstract] OR " Chi, Tai"[Title/Abstract] OR " Tai Chi Chuan"[Title/Abstract] OR "Taiji"[Title/Abstract] OR "Taijiquan"[Title/Abstract] OR "T'ai Chi"[Title/Abstract] OR "Tai Ji Quan"[Title/Abstract] OR "Ji Quan, Tai"[Title/Abstract] OR "Quan, Tai Ji"[Title/Abstract]  #2 "System"[Title/Abstract] OR "game"[Title/Abstract] OR "platform"[Title/Abstract] OR "online"[Title/Abstract]  #1 AND #2 AND ("2014/01/01"[Date - Publication] : "3000"[Date - Publication]) | 279 |
| Embase | #1 'tai chi'/exp OR 'tai ji':ti,ab,kw OR 'chi, tai':ti,ab,kw OR 'tai chi chuan':ti,ab,kw OR taiji:ti,ab,kw OR taijiquan:ti,ab,kw OR 'tai chi':ti,ab,kw OR 'tai ji quan':ti,ab,kw OR 'ji quan, tai':ti,ab,kw OR 'quan, tai ji':ti,ab,kw  #2 system:ti,ab,kw OR 'game':ti,ab,kw OR platform OR 'online':ti,ab,kw  #1 AND #2 AND [01-01-2014]/sd NOT [02-05-2024]/sd | 463 |
| Scopus | #1 ( TITLE-ABS-KEY ( Tai-ji ) OR TITLE-ABS-KEY ( Tai Chi ) OR TITLE-ABS-KEY ( Chi , Tai ) OR TITLE-ABS-KEY ( Tai Chi Chuan ) OR TITLE-ABS-KEY ( Taiji ) OR TITLE-ABS-KEY ( Taijiquan ) OR TITLE-ABS-KEY ( T'ai Chi ) OR TITLE-ABS-KEY ( Tai Ji Quan ) OR TITLE-ABS-KEY ( Ji Quan , Tai ) OR TITLE-ABS-KEY ( Quan , Tai Ji ) )  #2 ( TITLE-ABS-KEY ( System ) OR TITLE-ABS-KEY ( game ) OR TITLE-ABS-KEY ( platform ) OR TITLE-ABS-KEY ( online ) )  #1 AND #2 AND PUBYEAR > 2013 | 1,328 |
| IEEE Xplore | #1（"Abstract":tai ji) OR "Abstract":tai chi) OR ("Abstract":chi, tai) OR ("Abstract":tai chi chuan) OR ("Abstract":taiji) OR ("Abstract":taijiquan) OR ("Abstract":t'ai chi) OR ("Abstract":tai ji quan) OR ("Abstract":ji quan, tai) OR ("Abstract":quan, tai ji)）  #2 （"Abstract":system）OR （"Abstract":game）OR （"Abstract":platform）OR "Abstract":online）  #1 AND #2 AND Filters Applied: 01/01/2014 - 05/01/2024 | 120 |
| ACM Digital Library | #1 [Abstract: tai ji] OR [Abstract: tai chi] OR [Abstract: chi, tai] OR [Abstract: tai chi chuan] OR [Abstract: taiji] OR [Abstract: taijiquan] OR [Abstract: t'ai chi] OR [Abstract: tai ji quan] OR [Abstract: ji quan, tai] OR [Abstract: quan, tai ji]  #2 [Abstract: system] OR [Abstract: game] OR [Abstract: platform] OR [Abstract: online]  #1 AND #2 AND [E-Publication Date: (01/01/2014 TO 05/31/2024)] | 12 |
